# Vaccipack, A Mobile App to Promote Human Papillomavirus Vaccine Uptake Among Adolescents Aged 11 to 14 Years: Development and Usability Study

**DOI:** 10.2196/19503

**Published:** 2020-10-29

**Authors:** Anne M Teitelman, Emily F Gregory, Joshua Jayasinghe, Zara Wermers, Ja H Koo, Jennifer F Morone, Damien C Leri, Annet Davis, Kristen A Feemster

**Affiliations:** 1 University of Pennsylvania School of Nursing Philadelphia, PA United States; 2 Children’s Hospital of Philadelphia Philadelphia, PA United States; 3 Perelman School of Medicine University of Pennsylvania Philadelphia, PA United States; 4 Tufts University Medford, MA United States; 5 Yale University New Haven, CT United States; 6 Veterans Affairs West Haven, CT United States; 7 Big Yellow Star, Inc. Philadelphia, PA United States; 8 Division of Disease Control Department of Public Health Philadelphia, PA United States

**Keywords:** cervical cancer, prevention, mobile health, parents, adolescent health, vaccine, human papillomavirus

## Abstract

**Background:**

More than 90% of human papillomavirus (HPV)-related cancers could be prevented by widespread uptake of the HPV vaccine, yet vaccine use in the United States falls short of public health goals.

**Objective:**

The purpose of this study was to describe the development, acceptability, and intention to use the mobile app Vaccipack, which was designed to promote uptake and completion of the adolescent HPV vaccine series.

**Methods:**

Development of the mobile health (mHealth) content was based on the integrated behavioral model (IBM). The technology acceptance model (TAM) was used to guide the app usability evaluation. App design utilized an iterative process involving providers and potential users who were parents and adolescents. App features include a vaccine-tracking function, a discussion forum, and stories with embedded messages to promote intention to vaccinate. Parents and adolescents completed surveys before and after introducing the app in a pediatric primary care setting with low HPV vaccination rates.

**Results:**

Surveys were completed by 54 participants (20 adolescents aged 11 to 14 years and 34 parents). Notably, 75% (15/20) of adolescents and 88% (30/34) of parents intended to use the app in the next 2 weeks. Acceptability of the app was high among both groups: 88% (30/34) of parents and 75% (15/20) of adolescents indicated that Vaccipack was easy to use, and 82% (28/34) of parents and 85% (17/20) of adolescents perceived the app to be beneficial. Higher levels of app acceptability were found among parents with strong intentions to use the app (*P*=.09; 95% CI –2.15 to 0.15).

**Conclusions:**

mHealth technology, such as Vaccipack, may be an acceptable and nimble platform for providing information to parents and adolescents and advancing the uptake of important vaccines.

## Introduction

Human papillomavirus (HPV) is the most common sexually transmitted infection in the United States and a cause of cervical and other anogenital cancers [1]. HPV types 16 and 18 lead to 70% of cervical cancers, and HPV infection has been linked to anal, oral, and throat cancers in women and men [2]. HPV vaccines, available in the United States since 2006, are safe and highly effective [3]. The currently utilized 9-valent vaccine covers the HPV types associated with the majority of cervical, anal, and throat cancers and can prevent an estimated 92% of all HPV-related cancer cases [4]. Recent data demonstrate that HPV vaccines are associated with a significant reduction in precancerous lesions [5]. HPV vaccines are most beneficial when given to younger adolescents and before the onset of sexual activity. As such, the Advisory Committee on Immunization Practices recommends the HPV vaccine as a two-dose series for routine administration to both boys and girls between ages 11 and 12 years [6]. However, the vaccine may be given as early as age 9 and should be offered as catch-up to any adolescent through age 26.

Despite the profound individual and community-level benefits of HPV vaccination, rates in the United States consistently lag behind the other routinely recommended adolescent vaccines [7]. Recent data from the 2019 National Immunization Survey–Teen (NIS–Teen) [8] indicate that 72% of adolescents aged 13 to 17 years have received the first HPV dose and 54% have completed the age-appropriate HPV vaccine series compared with 90% for tetanus, diphtheria, and acellular pertussis vaccines and 89% for meningococcal vaccines. These data also show that there have been persistent differences in vaccine uptake across sociodemographic groups, including gender, insurance status, and community type. Higher series initiation rates have been observed in girls (68%) compared with boys (65%), adolescents covered by Medicaid (75%) and insurance (71%) compared with those uninsured (60%), and among adolescents who reside in urban (74%) and periurban (71%) areas compared with those from other/rural communities (64%) [8]. Periurban refers to areas outside of but near large metropolitan areas (populations greater than 50,000). In addition, White non-Hispanic adolescents have the lowest series initiation rate (68%) compared with peers from other racial and ethnic groups (71% to 77%) [8]. Lastly, NIS–Teen 2018 results demonstrated that among adolescents who had initiated the HPV vaccine series, completion rates were higher when parents received a provider recommendation (74.7%) than when there was no provider recommendation (46.7%) [9].

### Challenges to HPV Vaccine Uptake

Barriers to HPV vaccine uptake are well-described in the literature [10,11]. Understanding and addressing parental views about HPV vaccines may be critically important since parents/caregivers are the primary health care decision makers for their adolescents [12]. Many of these parental beliefs can inform behaviors around pursuing vaccines, consistent with the integrated behavioral model (IBM). IBM constructs have been widely used in identifying modifiable determinants of HPV vaccine behavior that are useful for informing interventions [13,14]. The IBM posits that intentions exert the most influence on behavior and, in turn, are influenced by attitudes, norms, and perceived behavioral control (also referred to as control or self-efficacy). Each of these three general constructs (attitudes, norms, and control) are influenced by a set of corresponding underlying beliefs. In relation to the HPV vaccine, behavioral beliefs refer to the favorable and unfavorable consequences, normative beliefs refer to those who would support or not support, and control beliefs refers to barriers and facilitators. Awareness and knowledge are considered relevant background factors that are necessary but often not sufficient for behavior change. Beliefs are potentially modifiable, and addressing these IBM social-cognitive constructs has led to many successful health behavior interventions [15]. Some known modifiable parental beliefs associated with HPV vaccine initiation or completion include (1) behavioral beliefs, such as vaccine safety and concern about sexual activity among adolescents [16-21], (2) normative beliefs, such as opinions of family, friends, and providers [17,19-28], (3) control beliefs, such as cost and access [16,28-30], (4) general views on vaccines, such as safety and effectiveness [20,31,32], and (5) knowledge and awareness of HPV and HPV vaccines [17,20,22,23,26,29,30,32-34] (see Multimedia Appendix 1 for a summary of these findings from the literature).

### Sources of Health Information

Parents rely heavily on their child’s health care providers to provide reminders for and schedule vaccines [35]. In recent years, there has been increased use of mobile phones to acquire health information, receive reminders, and track progress in attaining health goals. Patient-facing mobile health (mHealth) technology is useful to augment clinical care, as it can efficiently deliver information remotely before and after in-person clinic visits [36]. Additionally, smartphones are now accessible to a broad spectrum of the population [37]. Therefore, mHealth apps have great potential to deliver health behavior interventions to a broad spectrum of the US population.

As the use of mHealth technology expands, so too has the number of vaccine-focused health promotion initiatives, such as text messaging for reminders and recall appointments [38]. Studies examining the effectiveness of automated reminders and text messages to promote completion of the HPV vaccine series have yielded positive results [39-41]. There are a couple of mobile apps focused on helping parents understand adolescent development and health needs that include information on vaccines, although they have not yet been evaluated for impact [42,43]. There are also several apps available to help parents learn about and track recommended vaccines for infants and young children, but few include adolescent vaccines [44]. However, to our knowledge, no studies have evaluated the use of a mobile app to improve vaccine uptake through the dissemination of targeted educational messages that focus on the needs of young adolescents and support their emerging health care agency.

Thus, our objective was to develop an app (Vaccipack) as a tool to increase HPV vaccine acceptance by leveraging health behavior theory and mHealth to target key parental beliefs related to HPV vaccines. Vaccipack is exclusively focused on adolescent vaccines and designed for parents (to use and share with their adolescents) to promote the initiation and completion of the HPV vaccine series in their adolescent children.

Theory-based content design, although standard practice in behavioral intervention research, has not been a typical approach adopted by app developers [45]. Evaluation of acceptability and likely use, as we present here, is an important preliminary step for developing apps and in designing behavioral interventions that are most likely to achieve the desired health outcome [15,46]. Therefore, the purpose of this paper is (1) to describe the theory-based, community-informed development and features of the Vaccipack mobile app, and (2) to evaluate the acceptability of, and intention to use, the Vaccipack app among a sample of parents and parent-adolescent dyads presenting for a visit at a primary care clinic in which the HPV series’ initiation rate was lower than the national average.

## Methods

In this section, we first describe the development of the mobile app Vaccipack (phase 1a) and the features of the app that was created as a result of this process (phase 1b). Next, we describe the study that was conducted to evaluate the usability of Vaccipack (phase 2).

### Phase 1a: Mobile App Development

In developing Vaccipack content, we used the IBM-categorized parental beliefs about HPV vaccine uptake (see Multimedia Appendix 1) to increase motivation for uptake of the HPV vaccine [15]. We also incorporated basic information about HPV and the HPV vaccine. The app development process itself was also informed by applying user-centered design principles, which focused on designing an application from the perspective of the user [47]. Additionally, our preliminary evaluation of the app was guided by the technology acceptance model (TAM), which focuses on two aspects of acceptance—usability and usefulness—and the IBM, which indicates that intention is the most proximal predictor of behavior [48,49].

We elicited input from community advisory board (CAB) members. Adolescents and parents of adolescents were included as CAB members (as potential users), and pediatric clinicians were included in CAB meetings (as potential recommenders of the app). Five meetings with representatives from these CAB groups were held. Study team members facilitated each meeting using a discussion guide that focused on better understanding potential users’ current strategies for vaccine decision making, functions users might value in a vaccine-related app, and user preferences for potential app content. Wireframes (drawings of various functions as they would appear on screens) of the proposed app were also used to facilitate discussion in some of the meetings.

During each CAB meeting, we presented proposed app features and elicited recommendations for additional features. The name for the app, Vaccipack, was generated from the first adolescent CAB meeting and endorsed in a subsequent parent meeting. The adolescents indicated the name represented the ability to conveniently carry around the vaccine information, akin to a backpack. CAB members were not included in the participant dyads for the survey portion of this study.

Two of the central features of the Vaccipack app were to provide information about and tracking for all three recommended adolescent vaccines—HPV vaccine, Tdap (tetanus, diphtheria, and acellular pertussis) vaccine, and meningococcal conjugate vaccine—as well as the yearly influenza vaccine. While the focus of Vaccipack was to improve uptake of the HPV vaccine, it is important to strongly endorse all adolescent vaccines equally as a bundle [50]. Another central feature of Vaccipack was the use of brief stories to provide vaccine information and target modifiable parental beliefs associated with adolescents’ HPV vaccine uptake (Multimedia Appendix 1). To accomplish this, we created 26 stories addressing IBM constructs related to HPV vaccine behavior drawn from the literature as a way to promote HPV vaccine initiation and completion. Stories were written from the perspective of parents/primary caregivers, adolescents, and clinicians. Each story incorporated HPV and HPV vaccine knowledge and two to three HPV-related beliefs that might promote vaccine acceptance. Stories ranged from two to four paragraphs and were written at a middle school reading level. Topics were drawn from events that may occur in the daily lives of families with adolescents, including visits to see a health care provider. The 26 unique stories were developed so that a new weekly story could be highlighted with a push notification (indicated by a message on one’s mobile device noting, “you have a new story in Vaccipack”) for 26 weeks, or approximately 6 months, which is the amount of time needed to complete the two-dose HPV series for youth aged 11 to 14 years. If participants did not complete their HPV vaccine series in the 6-month time allotment, the series of 26 stories would repeat over the subsequent 6 months. Table 1 outlines the description of the five theory-derived concepts (based on the IBM) used as the framework for the content developed in each of the 26 stories: (1) behavioral beliefs, (2) normative beliefs, (3) control beliefs, (4) general norms, and (5) knowledge. The 26 stories were developed as two sets of 13 stories (set A and set B), with each set addressing all IBM theory-based modifiable factors at least once. Each theoretical concept addressed specific themes generated from the existing literature, and the relevant contextual content was drawn from CAB meetings. The frequency of each theme presented in the app story content is shown in Table 1.

Additionally, input obtained from our initial CAB meetings favored having an interactive component of the app; thus, a discussion forum was included as one of the app’s features. The app was collaboratively built by Big Yellow Star, Inc, with iterative feedback regarding content and functionality from members of the study team. The color scheme, illustrations for each story, and customized font were all designed to appeal to the target audience. In addition to the mHealth app, the developer also created a web-based staff console that allowed research staff to review forum posts and respond as needed.

**Table 1:** Frequency of human papillomavirus (HPV)-related themes addressed in 26 stories.

Theoretical concept	Theme	Set A (n=13)	Set B (n=13)	Total, n (%)
Behavioral belief	Vaccine safety and vaccine side effects	2	1	3 (12)
	Vaccine will not lead to increased sexual activity	2	3	5 (19)
	Cancer prevention	5	3	8 (31)
	Being a responsible parent	5	5	10 (38)
	Anticipated regret for not getting the vaccine	2	2	4 (15)
	Herd immunity (community benefit)	1	1	2 (8)
Normative belief	Provider recommendation	10	3	13 (50)
	Opinions of friends (positive and/or negative)	3	3	6 (23)
	Opinions of family (positive and/or negative)	2	1	3 (12)
Control belief	Cost	2	2	4 (15)
	Insurance coverage	2	2	4 (15)
	Access to provider	2	0	2 (8)
	Where to get vaccinated	2	0	2 (8)
General norm	General preventative care and immunizations	3	4	7 (27)
	Parent health history of HPV	2	1	3 (12)
	Family cultural background	2	1	3 (12)
	Media influence	0	1	1 (4)
Knowledge	Two doses versus three doses	3	2	5 (19)
	Do boys need the vaccine?	1	1	2 (8)
	What are the effects of HPV?	3	2	5 (19)
	How common is HPV?	3	3	6 (23)

### Phase 1b: Description of the Vaccipack Mobile App

Vaccipack is a free, downloadable mobile app for both iPhone operating system (iOS) and android formats that can be used on mobile phones and tablets. It is currently only available in English and is written for a middle school or higher reading level. A webpage for the app with some basic information is also available [15]. Vaccipack was designed to provide parents of adolescents aged 11 to 14 years with convenient access to vaccine health information. The application was designed to work in concert with provider recommendations and serves as a practical tool for parents (see Multimedia Appendix 2).

More specifically, the main features of the app include an introductory video, a vaccine checklist that serves a tracking function, stories about parents and their adolescents navigating adolescent vaccinations, and a discussion forum with additional resources on adolescent vaccines. A 2.5-minute video, displayed in a narrated animation format, provides basic information about meningococcal conjugate vaccine, Tdap vaccine, and HPV vaccine, as well as a reminder to get the yearly influenza vaccine. Ideally, a brief description of the app with instructions on how to download and view it would be available in the clinic waiting room, before seeing a provider. After the visit with a health care provider, the parent could enter information regarding vaccination histories for multiple children into the vaccine checklist for each adolescent child aged 11 to 14 years. Brief introductory information about each vaccine is also provided in the checklist. After the first HPV vaccine is entered, a set of push notification reminders are sent to the user (6, 9, and 12 months after the first dose) about the second dose (intended to be completed 6 to 12 months after the first dose). As described above, each story in the app addresses a few of the most common parental beliefs related to HPV vaccine uptake, such as provider recommendations, cost of the vaccine, and whether a vaccine should be delayed if the adolescent is not sexually active [9,10,13]. The stories describe families in realistic situations and address barriers to vaccine uptake and strategies to overcome them. Each story is followed by a list of several forum posts that are related to the topic of the story. On the forum, users can post new discussion items or respond to other posts. For each new forum post, the user can select one or more of the 14 “tags” to identify the topic, such as “vaccine schedule,” or “effects of HPV.” Users can filter the forum posts by these tags to read about topics they are most interested in. Several exemplar posts have been entered into the forum by members of the research team, consisting of several frequently asked questions with responses that include a mention of resources, such as the Vaccines for Children program, which provides financial assistance for vaccines to families in need [43].

### Phase 2: Acceptability and Intention-to-Use Evaluation of the Vaccipack App

To assess the acceptability of and intention to use the Vaccipack app, baseline and post-app surveys were designed to garner feedback from potential users. The baseline survey included items adapted from the Parent Attitudes About Childhood Vaccines survey instrument [51], technology acceptance model (TAM) [48,49], and the Pew Research Center’s health information survey [37]. Vaccine beliefs and behaviors included history of vaccine delay or refusal, trust in vaccine information, and belief that following the recommended immunization schedule is good for one’s child. Health information items included the source of health information parents and adolescents used, devices used to search for health information, comfort using mobile phones for health information, and current health app use. Responses were captured as discrete categories, measured yes/no/don’t know, agree/disagree, or using a Likert scale. A post-app introduction survey was also adapted from previously published work [52] and included items based on both the TAM [48,49] and the IBM. Survey items related to app acceptability included perceived usefulness and perceived ease of use. Items were rated on a 5-point Likert scale of agree/disagree. The three acceptability questions were combined into an acceptance measure, with a possible response range of 3 to 15. Items derived from the IBM [15] related to an intention to use the app in the next 2 weeks (5-point Likert scale of agree/disagree). In addition, there were four open-ended questions eliciting behavioral beliefs (eg, “What would be good/bad about using the app in the next 2 weeks?”) and self-efficacy beliefs (eg, “What would be easy/hard about using the app in the next 2 weeks?”). A final post-app survey item, scored on a 5-point Likert scale, asked, “How likely are you to share Vaccipack with your adolescent (parent survey) or parent (adolescent survey)?” Finally, app usage data were gathered in aggregate for participants and other users from Google Analytics.

### Eligibility and Recruitment

The app evaluation study protocol was reviewed and approved by the Institutional Review Board at Children’s Hospital of Philadelphia. Following approval, the study team enrolled parents and parent-adolescent dyads from a periurban primary care pediatric clinic. This primary care clinic was selected because its aggregate HPV vaccination rates among adolescents were below the national average. Dyad inclusion criteria were as follows: (1) the adolescent was between the ages of 11 and 14 years, (2) the adolescent had received primary care services at the study site, (3) the adolescent’s respective parent or guardian had a smartphone or tablet with Vaccipack app download capability, and (4) the adolescent and parent or guardian had the ability to read and understand English. Although the study aimed to recruit parent-adolescent dyads, parents could participate even if their adolescent was not interested in participating in the study. Parents or adolescents who participated in the app-development CAB group were not eligible to enroll in the survey study.

All eligible parents and parent-adolescent dyads were approached in the study site’s waiting room and, if interested in participation in the study, were consented and evaluated for eligibility. Recruitment occurred on specific study recruitment days over a 3-month period. Staff at the respective recruiting clinic ensured that we captured all eligible families. Consenting parents and adolescents individually completed a paper-and-pencil–based baseline survey that included sociodemographic characteristics (eg, age, race/ethnicity, education), immunization-related beliefs and behaviors, internet and app usage habits, and health information preferences. Following completion of the baseline survey, research staff introduced the Vaccipack app, which parents then downloaded. After an opportunity to review the app at the visit, parents and adolescents individually completed the second brief post-app-use paper-and-pencil–based survey.

### Data Analysis

Demographic data and closed-ended question responses from the pre- and post-app surveys were analyzed using descriptive statistics. Likert scale results were dichotomized due to skewed responses. An independent *t* test was used to assess the difference in mean values for the acceptability measure among parents, comparing those who strongly agreed (n=17) with those who did not strongly agree (n=17) that they intended to use the app in the next 2 weeks. Responses to the open-ended survey questions were coded by two research team members to identify dominant themes using the content analysis method described by Fishbein and Ajzen [15], which uses IBM concepts as the thematic framework. App usage data were summarized using descriptive statistics.

## Results

### Pre-App-Introduction Baseline Survey: Parents and Adolescents

Of the 34 parents who completed the parent baseline survey, 28 (83%) were aged 40 or older, 27 (79%) were White, 21 (62%) had completed a bachelor’s degree or higher, and 22 (65%) had a yearly household income of US $80,000 or more (Table 2). Eighteen of 34 (53%) parents kept track of their child’s vaccines using home records, while 9 of 34 (26%) parents relied on reminders from their child’s health care provider and 7 of 34 (21%) parents did not keep track of immunization records at all. Thirty-two of 34 (97%) parents thought that following the recommended vaccine schedule was a good idea for their child, and 29 of 34 (88%) parents indicated that they trusted the information they received about vaccines. However, of the 34 parents, 7 (21%) parents had delayed and 5 (15%) parents had declined having their child get a vaccine for reasons other than illness or allergy, with 9 (26%) parents endorsing one or both of these indicators of vaccine hesitancy.

In terms of health information–seeking practices for their own health, 30 of 34 (88%) parents relied on the internet and 27 of 34 (79%) parents relied on health care providers, while only 8 of 34 (24%) parents utilized health apps. Of the 34 parents, 24 (77%) had used a smartphone most frequently (compared with a computer or tablet) for seeking health-related information and 30 (88%) were comfortable doing so. Although only 1 of 34 (3%) parents had used an adolescent health app previously, 33 of 33 (100%) parents who responded believed that they could be a reliable source of information. Seventeen of 34 (50%) parents indicated that they would be very likely to use a health app if their health care provider recommended it, and 7 of 34 (21%) parents had privacy concerns. The most common anticipated barriers to using a teen health app that parents identified included cost of the app (14/34, 41%) and not having time to try it out (11/34, 32%) (see Multimedia Appendix 3).

Twenty adolescents, whose parents also participated in the study, completed the baseline adolescent survey. Adolescents were in grades 6 through 9. Eleven of 20 (55%) adolescents identified as girls and 9 of 20 (45%) identified as boys (Table 2). Most (13/20, 65%) of the adolescents relied on their parent or guardian for health information, with 50% (10/20) of adolescents indicating that the internet was their most common resource for seeking health information on their own.

**Table 2:** Parent and adolescent participants’ characteristics.

		Adolescents	Parents
Characteristic		(n=20), n (%)	(n=34), n (%)
**Parent age (years)**			
	32-39		6 (18)
	40-49		20 (59)
	50+		8 (24)
**Adolescent age (years)**			
	11-12	11 (55)	
	13-14	9 (45)	
**Race**			
	Black/African American	2 (10)	3 (9)
	White	13 (65)	27 (79)
	Other	5 (25)	4 (12)
**Hispanic/latino/latina ethnicity**			
	Yes	4 (20)	6 (18)
**Adolescent gender**			
	Female	11 (55)	
	Male	9 (45)	
**Parent education level**			
	Some high school/high school graduate/high school equivalency certificate		6 (18)
	Some college/technical training		7 (21)
	Bachelor’s degree		15 (44)
	Higher education degree		6 (18)
**Adolescent grade level**			
	Grade 6	4 (20)	
	Grade 7	10 (50)	
	Grade 8	4 (20)	
	Grade 9	2 (10)	
**Total household income in previous year (US $)**			
	$12,000-$24,000		2 (6)
	$25,000-$79,000		10 (29)
	$80,000 or higher		22 (65)
**Total household size**			
	3 people		8 (24)
	4 people		7 (21)
	5 people		12 (35)
	6 people		7 (21)

Seventeen of 20 (85%) adolescents reported that their parents made decisions about vaccines for them, while 3 of 20 (15%) adolescents made decisions together with their parents. Thirteen of 20 (65%) adolescents trusted the information they received about “shots,” while 6 of 20 (30%) adolescents were unsure that the information was accurate. Eleven of 20 (55%) adolescents were comfortable discussing health information with their parents, and 15 of 20 (75%) adolescents reported that they would receive a vaccine if recommended as being important by a parent or guardian (see Multimedia Appendix 4).

Although none of the 20 adolescents had previously used an adolescent health app, 19 (95%) indicated that they would be interested in using one if they learned about it from a doctor or nurse (whose endorsement they valued most) rather than from their parents (13/20, 65%), friends (9/20, 45%), or teachers (5/20, 25%). Adolescents stated that their trust in the information on an app would be higher if the app was recommended by doctors or nurses (15/20, 75%) than if recommended by parents (12/20, 60%) or friends (1/20, 5%). More than one-half (12/20, 60%) of adolescents indicated that an app would be a good way to learn about health information, and only 2 of 20 (10%) adolescents had privacy concerns. Potential barriers to using a health app included cost and not knowing how to use it (see Multimedia Appendix 5).

### Post-App-Introduction Survey: Parents and Adolescents

In total, 34 parents and 20 adolescents completed short surveys that were administered after participants had been introduced to the Vaccipack app and had had time to explore the features. Perceived ease of use was generally favorable for both groups, with more parents believing that Vaccipack was easy to learn about (31/34, 91%) and to use (30/34, 88%) compared with adolescents (14/20, 70% and 15/20, 75%, respectively). Regarding perceived usefulness, 28 of 34 (82%) parents and 17 of 20 (85%) adolescents agreed that Vaccipack would be beneficial. Of note, 15 of 20 (75%) adolescents and 30 of 34 (88%) parents intended to use the app in the next 2 weeks (Table 3). The measure of app acceptability among parents had a Cronbach alpha of .76 and an overall mean of 12.91 (range 9-15). The mean acceptability among parents who strongly agreed that they intended to use the app in the next 2 weeks (17/34, 50%) was 13.41 compared with 12.41 among parents who did not strongly agree (17/34, 50%), a difference that was marginally significant at *P*=.09 (95% CI 2.15 to 0.15).

Open-ended responses regarding anticipated app use benefits, drawbacks, barriers, and facilitators were similar for parents and adolescents. The most common reported benefits of the app were (1) being able to keep track of their (or their child’s) vaccine schedule, (2) being able to receive information about vaccines, and (3) being able to receive information about adolescents’ health. Most participants noted no drawbacks of the app. However, 2 of 20 (10%) adolescents mentioned that it would not be good if the app had “scary information.” Both adolescents and parents noted “time” as the most common anticipated barrier. Additionally, forgetting to check the app was a common potential barrier noted by parents, while not being sure of how to use the app was a common possible barrier for adolescents. Two of 20 (10%) adolescents also mentioned “lots of reading/a lot to get through” as an additional potential barrier. A common facilitator for app use among both parents and adolescents was the potential ease of use. In addition, parents cited having reminders and the convenience of having information readily available anywhere on their mobile device as potential facilitators of using the app. Finally, adolescents noted having been shown how to use the app as a significant facilitator.

**Table 3:** Post-app-introduction responses.

		Agree/strongly agree	
Concept/statement		Parents (n=34), n (%)	Adolescents (n=20), n (%)
**Acceptability—perceived ease of use**			
	Easy to learn how to use	31 (91)	14 (70)
	Easy to use	30 (88)	15 (75)
**Acceptability—perceived usefulness**			
	Will be beneficial	28 (82)	17 (85)
**Intention**			
	Likely to share with your teen	25 (74)	
	Likely to share with your parent/guardian		14 (70)
	Plan to use in next 2 weeks	30 (88)	15 (75)

### App Usage Indicators

App usage data specific to our study participants was available on the staff console for the three main features: tracking, stories, and forum. In the 2 weeks following enrollment, users accessed between one and three of the app’s features (mean two features): they read between one and 15 stories (mean three stories) and between one and 10 questions (mean three questions) in the forum. Although we did not advertise the app, it was free and publicly available on the app stores. According to Google Analytics, which provides overall app usage data, for the period from July 2018 to July 2019, there were 85 users (15 were research staff and developers), most of who lived in the greater Philadelphia area. It was also accessed in several US cities (eg, Washington, New York, Houston, and Cupertino), and it was viewed in China and Sweden. Most of the individuals who accessed the app used the iOS (Apple) version (70/85, 82%), with the remaining 18% (15/85) using the android version.

## Discussion

### Principal Results

The Vaccipack app was designed to be used primarily by parents, but it was also meant to be shared with adolescents as an avenue for promoting adolescent involvement in their own health care. Intention to use Vaccipack was high among both parents and adolescents after they had been introduced to the app and had some time to explore it, although it was higher among parents (30/34, 88%) than among teens (15/20, 75%). Perhaps fewer adolescents viewed the app as relevant to them given that 85% (17/20) indicated their parents made the decisions about their vaccines. In addition, most parents and adolescents found Vaccipack easy to use. However, a higher percentage of parents (31/34, 91%) found the Vaccipack app easy to learn how to use compared with adolescents (14/20, 70%). This could be due to adolescents not having used a similar app before, a reason indicated by 35% (7/20) of adolescents as an impediment to learning how to use a teen health app, which by comparison was only endorsed as a potential barrier by 18% (6/34) of parents. Perhaps adolescents needed more time to explore the app to become comfortable using it. Notably, higher levels of app acceptability were found among parents with strong intentions to use the app compared with others, an association that trended toward significance. These findings are encouraging given that previous studies using the TAM to examine various forms of technology use have found a strong relationship between both components of acceptability (perceived usefulness and perceived ease of use), intention to use, and actual use [48]. In developing technology-based interventions, it is important to first assess their acceptance among the target population, as we have done in this study.

Given as open-ended responses, the most common anticipated benefits of Vaccipack noted by parents and adolescents were to help keep track of required vaccinations and to access information on adolescent vaccines and health more generally. These findings support the relevance of the main features of the app—the vaccine tracker, video, stories, and forum. Open-ended responses from parents and adolescents suggest that an app that is easy to use, not complicated to learn, does not require too much reading, and provides easy-to-find information would facilitate its use. To further refine Vaccipack, future iterations could turn the written stories into audio or short video versions to make them more engaging and accessible [53]. Actual Vaccipack app use in the 2 weeks following the study visit varied widely, with a mean usage of two features—accessing three stories and three forum posts—which was very favorable given that the app was designed to highlight one story per week.

Our sample consisted of 34 parents (79% White) and 20 adolescents aged 11 to 14 years in a periurban setting, with two-thirds (22/34, 65%) of participating families having yearly household incomes of US $80,000 or higher (likely with private insurance). While almost all parents thought it was a good idea to adhere to the recommended vaccine schedule, more than one-quarter (9/34, 26%) of parents endorsed one or two indicators of vaccine hesitancy. Given that our study’s participants were from a clinic with HPV vaccination rates below the national average, these findings echo national statistics that indicate children aged 11 to 14 years whose families have private insurance have lower HPV vaccination rates compared with those covered by Medicaid [8]. Across race/ethnicity groups, the rates of HPV vaccination initiation and completion are lowest among White non-Hispanic families compared with all other groups [8].

Approximately one-half of the parents in this study did not keep track of their adolescent’s vaccines, and most relied on their provider or clinic to provide reminders for upcoming vaccines. This may help explain why parents may not be fully aware of or overestimate their adolescent’s vaccination status [35], and it suggests that an app, such as Vaccipack, with vaccine tracking and reminder features may be beneficial. As adolescents take on more responsibility for monitoring and maintaining their vaccine records, Vaccipack could be a helpful adjunct to support that process. It is notable that adolescents indicated they would be more likely to use an app and trust its content if they heard about it from a health care provider rather than from their parents or friends. This suggests providers may have a window of opportunity to talk about health technology use directly with young adolescent patients.

HPV vaccines are highly effective at preventing cancer, yet even with successful biologic interventions, the advantages will be limited if they are not adopted by those who can benefit. Thus, vaccine uptake behavior is an important area of study, especially now with promising COVID-19 vaccines on the horizon along with substantial skepticism of these new biologics [54,55]. mHealth is a promising area with the ability to promote advantageous health behaviors, such as vaccine uptake, especially because information can reach families remotely, but it will be vital to build apps on a strong foundation of behavioral science in order to be effective. For that reason, it will be important to guide content using well-validated health behavior theories, such as the IBM [15], which was used to create Vaccipack. Additionally, theory-guided interventions enable the measurement and evaluation of relevant mediators, such as beliefs, represented in component messages, which are the levers of behavior change [15]. Identifying relevant mediators can also assist in streamlining app content to include the most essential elements.

Patient-facing apps and mobile technologies provide a nimble platform to modify messages to parents and adolescents as scientific understanding of diseases, vaccines, and factors influencing vaccination evolves. In addition, the future potential for compatibility of such apps with patient portals and electronic health records will facilitate bidirectional access to relevant data, such as vaccine records, which will make apps like Vaccipack both easier to use (eg, automated data entry) and more useful (eg, able to download verified vaccine records into the app to share with schools, etc).

In order to advance patient-focused interventions to promote uptake of the HPV vaccine during the adolescent years, the Vaccipack app was designed for parents and their adolescents aged 11 to 14 years to provide information, assist with vaccine tracking, and enhance motivation for all adolescent vaccines, with more detailed content specifically on the initiation and completion of the HPV vaccine series. The longer-term goal of Vaccipack as an intervention is to increase knowledge and favorable outcome expectancies, promote norms of vaccine acceptance, and support self-efficacy by building skills and providing resources for HPV vaccine initiation and completion among parents for their vaccine-eligible adolescents because parents are the primary decision makers for younger adolescents. However, as adolescents are beginning to develop more agency related to their own health care [56], the app was secondarily developed to directly engage adolescents who may be involved in learning about their own health. Although this app is focused on the parent and their adolescent, and is not a provider-focused app, another goal of Vaccipack was to provide additional support to providers before and after the primary care visit. Undoubtedly, a strong provider recommendation for the HPV vaccine is among the strongest influencing factors for vaccine uptake [9]. For example, the introductory video in Vaccipack, which contains basic vaccine information, was intended to be viewed before seeing the health care provider, who could then focus on providing patient-centered decision-making support regarding vaccine uptake.

### Limitations

This research has a few limitations that can be addressed in future studies. The sample was drawn from one periurban clinic in the northeastern United States, which may not represent the variety of settings with lower vaccine uptake. This limits generalizability, and further research is needed that encompasses a variety of settings in various geographical regions, notably in rural areas where HPV vaccination rates are also low [8]. This is especially needed because national statistics indicate that some differences in smartphone ownership persist by location as well—83% ownership in urban and suburban residents, and 71% in rural residents [37]. Because we recruited in a health care facility, this study did not examine access to care among those who are uninsured, which is also an important barrier to HPV vaccine uptake given that those without insurance have among the lowest rates of HPV vaccine initiation [8]. The age range targeted for vaccine promotion was limited to adolescents aged 11 to 14 years. This was because behavioral interventions with a focused target behavior and group can be assessed more effectively [15]. However, older adolescents could also benefit from an app, such as Vaccipack, but would likely need a different type of app given the greater autonomy they have over their health care. Future app development and evaluation research could include participants with greater income, race and ethnic diversity, geographic diversity, and a wider age range of adolescents. While our current findings are promising, future research is needed to assess the actual use of the Vaccipack app in a larger sample and over time and its impact on HPV vaccine uptake behavior.

### Conclusions

Incorporating user-centered design principles led to the development of an app to promote the HPV vaccine that was acceptable in a sample of parents and adolescents in a clinic with lower HPV vaccination rates than the national average. Our theory-based approach to content development will facilitate future evaluation. It is remarkable that HPV vaccines can prevent many HPV-related cancers, yet tragic that this valuable vaccine has suboptimal uptake in the US. mHealth technology, such as Vaccipack, may provide an acceptable and nimble platform to provide information to parents and adolescents and advance uptake of life-saving vaccines.

## Appendix

Multimedia Appendix 1 Parental social-cognitive factors associated with adolescent’s HPV vaccine uptake identified in the literature.

Multimedia Appendix 2 Vaccipack app screenshots.

Multimedia Appendix 3 Parent health technology use and perspectives on adolescent health applications (n=34).

Multimedia Appendix 4 Adolescent vaccine practices (n=20).

Multimedia Appendix 5 Adolescent health-related technology use (n=20).

## Abbreviations

CAB: community advisory board
HPV: human papillomavirus
IBM: integrated behavioral model
mHealth: mobile health
NIS–Teen: National Immunization Survey–Teen
TAM: technology acceptance model
Tdap: tetanus, diphtheria, and acellular pertussis
