# Supplementary material for: Vaccipack, A Mobile App to Promote Human Papillomavirus Vaccine Uptake Among Adolescents Aged 11 to 14 Years: Development and Usability Study
Source: JMIR Nurs. 2020 Oct 29;3(1):e19503. doi: 10.2196/19503 (PMC8279454; doi:10.2196/19503)
Supplement: Multimedia Appendix 1 [file nursing_v3i1e19503_app1.docx]

| **Construct: Specific beliefs or other cognitive factors related to HPV vaccine** |
| --- |
| **Behavioral (attitudinal) beliefs (favorable and unfavorable consequences)** |
| - Vaccine Safety - Vaccine Side effects - Vaccine will /will not lead to adolescent’s increased sexual activity - Cancer prevention: vaccine will help prevent cancer - Being a responsible parent   - E.g.: I am being a responsible parent by getting my child vaccinated - Anticipated regret not getting the vaccine - Herd immunity - community benefit |
| **Normative beliefs (who would support/not support)** |
| - Provider recommendation - Positive or negative opinions of friends   - E.g.: Other parents are/are not vaccinating their kids at a younger age (not needed now) - Positive or negative opinions of family - Knowing someone with a history of HPV or cervical cancer |
| **Control beliefs (barriers and facilitators)** |
| - Cost - Insurance coverage - Access to provider - Knowing where to get vaccinated |
| **Beliefs about vaccines in general** |
| - Vaccines are/are not effective preventive care - Positive or negative influence of media sources on vaccination |
| **Awareness & knowledge of HPV and HPV vaccine** |
| - HPV awareness/lack of awareness - HPV vaccine awareness/lack of awareness |
| - Short term and long-term effects of HPV infection - Prevalence of HPV |
| - Two doses vs three doses:   - E.g.: Two doses when younger, vs three doses later - Boys and girls both need the vaccine - Impact of completing series over a longer period of time than recommended |
