# Supplementary material for: Vaccipack, A Mobile App to Promote Human Papillomavirus Vaccine Uptake Among Adolescents Aged 11 to 14 Years: Development and Usability Study
Source: JMIR Nurs. 2020 Oct 29;3(1):e19503. doi: 10.2196/19503 (PMC8279454; doi:10.2196/19503)
Supplement: Multimedia Appendix 2 [file nursing_v3i1e19503_app2.pdf]

Verizon 9:36 PM

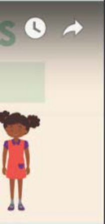

e 6-12...

- Home
- Stories
- Forum
- Checklist

MORE

- Video
- Profile
- About
- Log Out

vaccipack

Welcome to Vaccipack.

This app is designed to help you keep track of your vaccination history.

LOG IN

NEW ACCOUNT

Verizon 6:24 PM

Home

INTRO VIDEO

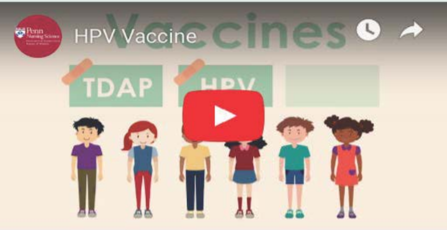

VACCINES NEEDED

HPV 2nd dose

For **Ben**

Don't forget to get the the 2nd dose 6-12...

HPV 1st dose

For **Alex**

Verizon 9:37 PM

Forum

CREATE A NEW POST

This is a forum for users to start a discussion, exchange tips and ask questions. An expert may weigh in.

All ▾

Waiting until marriage

Tags: Increased sexual activity , Age of vaccination , How common is HPV? , Family history of HPV , Do boys need it?

17 Sep

0

Can my child get infected with HPV if they wait until they get married to have sex? **READ MORE** ➡
