# Supplementary material for: Vaccipack, A Mobile App to Promote Human Papillomavirus Vaccine Uptake Among Adolescents Aged 11 to 14 Years: Development and Usability Study
Source: JMIR Nurs. 2020 Oct 29;3(1):e19503. doi: 10.2196/19503 (PMC8279454; doi:10.2196/19503)
Supplement: Multimedia Appendix 3 [file nursing_v3i1e19503_app3.docx]

| Use of Technology for health-related content |  | N | % ^a^ |
| --- | --- | --- | --- |
|  |  |  |  |
| **What sources do you use for seeking health information for yourself? (check all that apply)** |  |  |  |
|  | Internet | 30 | 88% |
|  | Books/Magazines | 12 | 35% |
|  | Friends/Family members | 14 | 41% |
|  | Health related mobile applications | 8 | 24% |
|  | Health care providers | 27 | 79% |
| **What sources do you use for seeking health information for your teen? (check all that apply)** |  |  |  |
|  | Internet | 26 | 76% |
|  | Books/Magazines | 9 | 26% |
|  | Friends/Family members | 15 | 44% |
|  | Health related mobile applications | 9 | 26% |
|  | Health care providers | 30 | 88% |
| **Most frequently used device to access the internet for health-related information (n=31)** |  |  |  |
|  | Computer | 5 | 16% |
|  | Tablet | 2 | 6% |
|  | Smart Phone | 24 | 77% |
| **Have you used a mobile phone to search for health-related information for your teen in the past year?** |  |  |  |
|  | Yes | 30 | 88% |
| **I am comfortable using a mobile phone to seek health related information for my teen** |  |  |  |
|  | Strongly agree/Agree | 30 | 88% |
|  | Neutral | 2 | 6% |
|  | Disagree/Strongly disagree | 2 | 6% |
| **Do you use any teen health apps?** |  |  |  |
|  | Yes | 1 | 3% |
| **How likely are you to use a teen health app on your mobile phone if your health care provider recommended it?** |  |  |  |
|  | Very likely | 17 | 50% |
|  | Likely | 13 | 38% |
|  | Somewhat likely | 4 | 12% |
| **Do you have any privacy concerns about using a teen a health app?** |  |  |  |
|  | Yes | 7 | 21% |
| **Do you think teen health apps can be reliable source of information? (n=33)** |  |  |  |
|  | Yes | 33 | 100% |
| **It would be hard to use a teen health app on my mobile phone if (check all that apply)** |  |  |  |
|  | I had not used a similar application before | 6 | 18% |
|  | If I was not shown how to use it | 6 | 18% |
|  | If I did not have time to try it out | 11 | 32% |
|  | If there was a charge and I could not afford it | 14 | 41% |
|  | Other: It used a lot of data/Not enough memory or storage | 2 | 6% |
| ^a^ % rounded to the nearest whole number | | | |
