# Supplementary material for: Vaccipack, A Mobile App to Promote Human Papillomavirus Vaccine Uptake Among Adolescents Aged 11 to 14 Years: Development and Usability Study
Source: JMIR Nurs. 2020 Oct 29;3(1):e19503. doi: 10.2196/19503 (PMC8279454; doi:10.2196/19503)
Supplement: Multimedia Appendix 4 [file nursing_v3i1e19503_app4.docx]

| Characteristic |  | N | % ^a^ |
| --- | --- | --- | --- |
|  |  |  |  |
| **Who makes decisions whether you get vaccines?** |  |  |  |
|  | My parents/guardians do | 17 | 85% |
|  | My parents/guardians and I make decisions together | 3 | 15% |
|  | *Options not selected: I do; my school does; I don’t know; someone else |  |  |
| **I trust the information I receive about shots** |  |  |  |
|  | I agree | 13 | 65% |
|  | I disagree | 1 | 5% |
|  | I’m not sure | 6 | 30% |
| **I am comfortable discussing teen health information with my parent/guardian** |  |  |  |
|  | Yes, definitely | 11 | 55% |
|  | Maybe | 6 | 30% |
|  | Probably not | 1 | 5% |
|  | No | 1 | 5% |
| **I am more likely to get all the vaccines that are recommended to me if my parent/guardian thinks vaccines are important** |  |  |  |
|  | Yes, definitely | 15 | 75% |
|  | Maybe | 3 | 15% |
|  | Probably not | 1 | 5% |
| ^a^ % rounded to the nearest whole number | | | |
