# Supplementary material for: Vaccipack, A Mobile App to Promote Human Papillomavirus Vaccine Uptake Among Adolescents Aged 11 to 14 Years: Development and Usability Study
Source: JMIR Nurs. 2020 Oct 29;3(1):e19503. doi: 10.2196/19503 (PMC8279454; doi:10.2196/19503)
Supplement: Multimedia Appendix 5 [file nursing_v3i1e19503_app5.docx]

|  |  | N | % ^a^ |
| --- | --- | --- | --- |
| **Do you use any teen health app?** |  |  |  |
|  | Yes | 0 |  |
| **I would be interested in using a teen health app if I heard it from... Yes, definitely/maybe** |  |  |  |
|  | My parents/guardians | 13 | 65% |
|  | My teachers | 5 | 25% |
|  | My doctors/nurses | 19 | 95% |
|  | My friends | 9 | 45% |
|  | On the TV or the internet | 1 | 5% |
| **I would trust information I got from a teen health app if I heard it from... yes, definitely** |  |  |  |
|  | My parents/guardians | 12 | 60% |
|  | My teachers | 5 | 25% |
|  | My doctors/nurses | 15 | 75% |
|  | My friends | 1 | 5% |
|  | On the TV or not the internet | 1 | 5% |
| **I think a teen health app would be a good way to learn about health information** |  |  |  |
|  | Yes, definitely | 12 | 60% |
|  | Maybe | 7 | 35% |
|  | Probably not | 1 | 5% |
| **Do you have any privacy or safety concerns about using a teen health app?** |  |  |  |
|  | Yes | 2 | 10% |
| **It would be hard to use a teen health app on my mobile phone if (check all that apply)** |  |  |  |
|  | I had not used a similar application before | 7 | 35% |
|  | I was not shown how to use it | 8 | 40% |
|  | I did not have time to try it out | 8 | 40% |
|  | There was a charge and I could not afford it | 10 | 50% |
|  | Other: I do not own a phone/I would eventually not use it | 2 | 10% |
| ^a^ % rounded to the nearest whole number | | | |
